# Genome-Wide Analysis of Alpharetroviral Integration in Human Hematopoietic Stem/Progenitor Cells

**DOI:** 10.3390/genes5020415

**Published:** 2014-05-16

**Authors:** Arianna Moiani, Julia Debora Suerth, Francesco Gandolfi, Ermanno Rizzi, Marco Severgnini, Gianluca De Bellis, Axel Schambach, Fulvio Mavilio

**Affiliations:** 1 Genethon, 1bis Rue de l’Internationale, 91020 Evry, France; E-Mails: amoiani@genethon.fr (A.M.); fgandolfi@genethon.fr (F.G.); 2 Institute of Experimental Hematology, Hannover Medical School, Carl-Neuberg-Str.1, D-30625 Hannover, Germany; E-Mails: suerth.julia@mh-hannover.de (J.D.S.); schambach.axel@mh-hannover.de (A.S.); 3 Institute for Biomedical Technologies, Consiglio Nazionale delle Ricerche, Milan 20132, Italy; E-Mails: ermanno.rizzi@itb.cnr.it (E.R.); marco.severgnini@itb.cnr.it (M.S.); gianluca.debellis@itb.cnr.it (G.B.)

**Keywords:** retroviral integration, alpha-retroviral vectors, gene therapy, human hematopoietic cells

## Abstract

Gene transfer vectors derived from gamma-retroviruses or lentiviruses are currently used for the gene therapy of genetic or acquired diseases. Retroviral vectors display a non-random integration pattern in the human genome, targeting either regulatory regions (gamma-retroviruses) or the transcribed portion of expressed genes (lentiviruses), and have the potential to deregulate gene expression at the transcriptional or post-transcriptional level. A recently developed alternative vector system derives from the avian sarcoma-leukosis alpha-retrovirus (ASLV) and shows favorable safety features compared to both gamma-retroviral and lentiviral vectors in preclinical models. We performed a high-throughput analysis of the integration pattern of self-inactivating (SIN) alpha-retroviral vectors in human CD34^+^ hematopoietic stem/progenitor cells (HSPCs) and compared it to previously reported gamma-retroviral and lentiviral vectors integration profiles obtained in the same experimental setting. Compared to gamma-retroviral and lentiviral vectors, the SIN-ASLV vector maintains a preference for open chromatin regions, but shows no bias for transcriptional regulatory elements or transcription units, as defined by genomic annotations and epigenetic markers (H3K4me1 and H3K4me3 histone modifications). Importantly, SIN-ASLV integrations do not cluster in hot spots and target potentially dangerous genomic loci, such as the EVI2A/B, RUNX1 and LMO2 proto-oncogenes at a virtually random frequency. These characteristics predict a safer profile for ASLV-derived vectors for clinical applications.

## 1. Introduction

Transplantation of hematopoietic stem cells genetically modified by retroviral vectors has proven its clinical efficacy in a number of seminal clinical trials for the treatment of severe monogenic disorders [1,2,3,4,5,6,7,8]. However, some of these studies also showed the genotoxic risks associated with the insertion of foreign DNA in the human genome, which limit the clinical application of integrating vectors (reviewed in [9]). Several efforts have been made to improve the safety of retroviral vectors, leading to the design of safer constructs and the development of robust *in vitro* and *in vivo* genotoxic assays to predict the potential risk associated with their integration into the genome [10,11,12]. High-definition mapping of integration sites of vectors derived from the Moloney murine leukemia virus (MLV) and human immunodeficiency virus (HIV) in murine and human cells revealed non-random profiles with a strong tendency to target active regulatory regions for MLV-derived gamma-retroviral vectors [13,14] and transcribed regions for HIV-derived lentiviral vectors [15,16]. These integration patterns explain the relatively high risk to deregulate gene expression at the transcriptional or post-transcriptional level observed in pre-clinical, as well as in clinical studies (reviewed in [9]).

Small-scale surveys of integration sites of vectors derived from alpha-retroviruses, such as the avian sarcoma-leukosis virus (ASLV), in different cell types indicated a more random pattern compared to other retroviruses, with a slight preference for transcription units, but no apparent preference for promoters and transcription start sites (TSSs) [17,18,19,20]. This potentially more favorable integration profile prompted the development of a replication-deficient, self-inactivating (SIN) ASLV-derived vector capable of efficiently transducing murine and human cells [21]. This vector was able to sustain long-term transgene expression in murine and human hematopoietic progenitors at levels comparable to those obtained with SIN-MLV and SIN-HIV vectors and to correct the X-linked chronic granulomatous disease (X-CGD) phenotype in a mouse model of the disease [20,22].

We and others previously reported that MLV, SIN-MLV and SIN-HIV integrations are highly clustered in the human genome, with cell-specific patterns that correlate with the transcriptional program and the epigenetic landscape of each cell type [14,15,16,19,23,24,25,26]. In this study, we report a high-definition analysis of the integration patterns of SIN-MLV, SIN-ASLV and SIN-HIV vectors in human CD34^+^ hematopoietic stem/progenitor cells (HSPCs), which was carried out to evaluate their comparative genotoxic potential in a clinically relevant target cell. We show that the SIN-ASLV integration profile is close to random, with no preferential targeting of TSSs or transcribed genes compared to SIN-MLV and SIN-HIV. The SIN-ASLV vector does not target CpG islands, conserved non-coding regions (CNCs) or elements enriched in transcription factor binding sites (TFBS), is less frequently associated with epigenetically defined promoter and enhancer regions compared to SIN-MLV and is randomly associated with repetitive elements in the genome. Similarly, we observed no preference for transcribed regions compared to SIN-HIV. Heterochromatic regions are excluded by the integration pattern of all three vectors. Interestingly, the ASLV vector showed no apparent clustering in the genome and has no association with the typical integration hot spots observed for MLV- and HIV-based vectors. These results highlight a safer integration profile of alpha-retroviral vectors in human cells, supporting their development as a clinical gene transfer tool.

## 2. Experimental

### 2.1. Vectors and Cells

Human CD34^+^ HSPCs were purified form umbilical cord blood, pre-stimulated for 48 h in serum-free Iscove’s modified Dulbecco medium supplemented with 20% Fetal Calf Serum (FCS), 20 ng/mL human thrombopoietin, 100 ng/mL Flt-3 ligand, 20 ng/mL interleukin-6 and 100 ng/mL stem cell factor, as previously described [23]. HSPCs were transduced with the SIN-ASLV vector, pAlpha.SIN.EFS.EGFP.WPRE (noTATA), expressing GFP under the control of the elongation factor 1α promoter, pseudotyped in an amphotropic envelope by three-plasmid transfection in 293T cells, as previously described [20]. Cells were infected by 3 rounds of spinoculation (1500 rpm for 45 min) in the presence of 4 μg/mL polybrene. Transduction efficiency was evaluated by cytofluorimetric analysis of GFP expression 48 h after infection.

### 2.2. Amplification, Sequencing, and Analysis of Retroviral Integration Sites

Genomic DNA was extracted from a pool of 3.5 × 10^6^ CD34^+^/GFP^+^ cells enriched by fluorescence-activated cell sorting, after a brief period in culture to dilute unintegrated vectors. 3'-LTR vector-genome junctions were amplified by LM-PCR adapted to the GS-FLX Genome Sequencer (Roche/454 Life Sciences) pyrosequencing platform, as previously described [14]. Raw sequence reads were processed by an automated bioinformatic pipeline that eliminated small and redundant sequences [14] and mapped on the University of California at Santa Cruz (UCSC) hg19 release of the human genome [14]. All UCSC RefSeq genes having their TSS at ±50 kb from an integration site were annotated as targets. Genomic features were annotated when their genomic coordinates overlapped for ≥1 nucleotide with a ±1 kb interval around each integration site. We used UCSC tracks for both CpG islands and conserved TFBSs, and the previously described genomic coordinates of 82,335 mammalian conserved non-coding sequences (CNCs) [27]. Raw sequences having a single or ambiguous match in the genome (the latter mapping in multiple genomic positions with a difference in the identity <2) were blasted on the UCSC RepeatMasker database. DNase I hypersensitive sites from publicly available data [28] were annotated when overlapping for at least 1 bp with a ±1-kb interval around an integration. Repetitive elements were annotated when directly targeted by each integration site. Sequences having multiple matches were collapsed and counted as one when matching in the same genomic positions and were univocally associated with the single type of repetitive element they targeted.

For the association of the integrations with epigenetically defined chromatin states, we used publicly available ChIP-Seq data (NIH Roadmap Epigenomics Mapping Consortium database) that we re-annotated in the UCSC hg19 release of the human genome. We analyzed the distribution of integration sites around histone modifications (H3K4me1, H3K4me3, H3K36me3, H3K27me3) using the seqMINER platform [29]. Previously generated SIN-MLV, SIN-HIV integrations and random control sequences datasets [14] were also re-annotated on the UCSC hg19 genome. For all pairwise comparisons, we applied a 2-sided Fisher’s exact test. The threshold for statistical significance was set at a *p*-value < 0.01.

## 3. Results and Discussion

### 3.1. SIN-ASLV Vectors Exhibit an Almost Random Integration Profile in the Genome of Human CD34^+^ HSPCs

To generate a high-definition alpha-retroviral integration profile in human HSPCs, we transduced umbilical cord blood-derived CD34^+^ cells with a previously described SIN-ASLV vector carrying a GFP expression cassette under the control of the intron-less, 240-bp version of the elongation factor-1α (EFS) promoter [20]. Cells were transduced at 10% to 20% efficiency and were selected for GFP expression by cell sorting 10 days after infection, to dilute unintegrated vectors. Vector-genome junctions were amplified from genomic DNA by ligation-mediated (LM)-PCR and pyrosequenced, as previously described [14]. Raw sequences (available at GenBank with the accession number SRR1282019) were processed by a previously described bioinformatic pipeline [14] and mapped on the UCSC hg19 release of the human genome, to obtain 8250 unique insertion sites. Two datasets of SIN-MLV (13,097) and SIN-HIV (31,827) vector integrations, previously generated in human umbilical cord blood-derived CD34^+^ cells in comparable experimental conditions, and a set of in-silico generated normalized random sites (40,000) [14,26] were re-annotated on the hg19 genome and used for comparison. To identify differences in the integration preferences of SIN-ASLV compared to SIN-MLV and SIN-HIV in human HSPCs, we first analyzed the distribution of integration sites around RefSeq genes in the human genome: integration was annotated as TSS-proximal when occurring in an interval of ±2.5 kb from the TSS of any RefSeq gene, intragenic when occurring inside a RefSeq gene >2.5 kb from the TSS and intergenic in all other cases.

**Figure 1:**
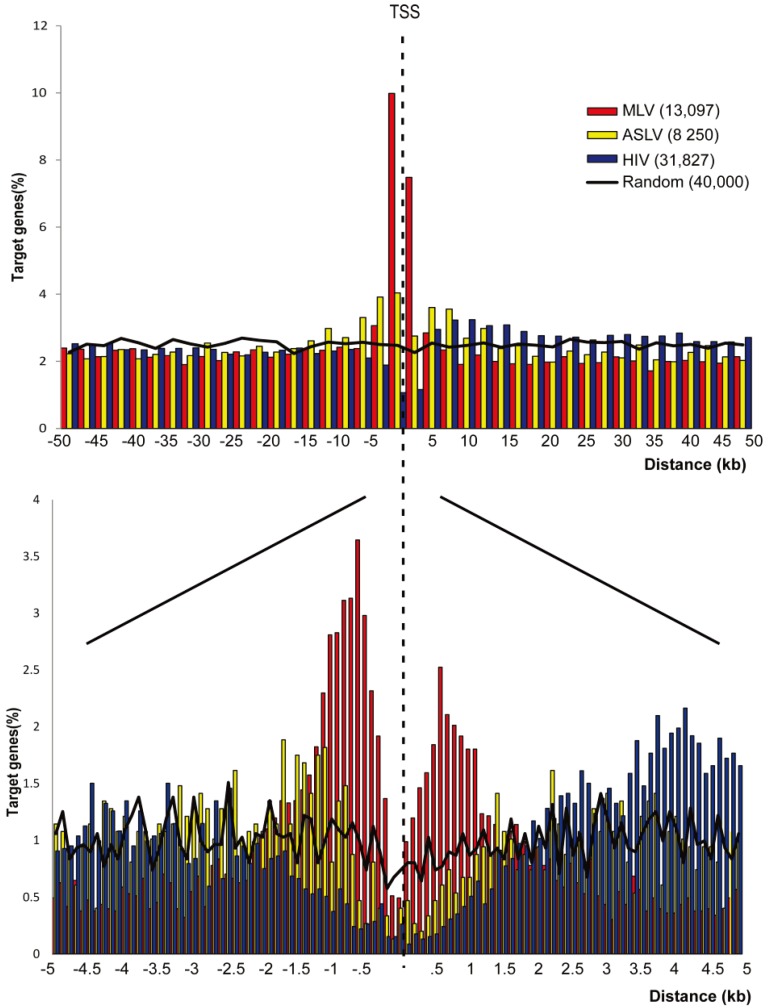
Genomic distribution of SIN-MLV, SIN-ASLV and SIN-HIV integrations in human HSPCs. The distribution of the distance of SIN-MLV (red bars), SIN-ASLV (yellow bars) and SIN-HIV (blue bars) integration sites from the TSS of targeted genes at 2500-bp (**a**) or 50-bp (**b**) resolution. The percentage of genes targeted at each position is plotted on the y-axis. The black line indicates the distribution of random control sites.

The high-definition profile of SIN-ASLV integration showed only a modest preference for TSSs (6.97% of the integration sites were annotated as TSS-proximal) compared to SIN-HIV and random sites (3.45% and 3.16%, respectively), which was significantly lower than that observed for the SIN-MLV vector (23.38%, *p* < 0.01). Similarly, SIN-ASLV showed only a slight tendency to integrate into genes (49.48% *vs.* 40.58% of random sites), significantly lower than that observed for SIN-HIV vectors (76.77%, *p* < 0.01). As a consequence, the frequency of SIN-ASLV integration outside transcription units was only slightly lower than random (43.55% *vs.* 56.26%) and significantly higher than those observed for the other two vectors (34.36% and 19.78%, respectively, *p* < 0.01) (Table 1). A plot of the relative distance of SIN-ASLV integration sites in an interval of ±50 kb from any TSS revealed a spread distribution with only a modest accumulation in the ±2.5 kb interval around TSS compared to the SIN-MLV vector. A higher definition map (100-bp intervals) showed the absence of integrations in the basal promoter region, most likely occupied by the RNA PolII basal transcriptional machinery. Integrations of the SIN-HIV vector were under-represented in a much wider interval of ±2.5-kb around the TSS (Figure 1).

**Table 1:** Integration distribution around RefSeq genes and genomic features in the genome of human hematopoietic stem/progenitor cells (HSPCs).

Vector	Intergenic (%)	TSS-proximal (%)	Intragenic (%)	CpG islands (%)	CNCs (%)	TFBS (%)	Total integrations
**SIN-ASLV**	43.55 *	6.97	49.48	2.84	5.49	55.70	8250
**SIN-MLV**	34.36 *	23.38 *	42.26	17.68 *	8.42	69.95 *	13,097
**SIN-HIV**	19.78 *	3.45	76.77 *	1.23	4.58	54.61	31,827
**Random**	56.26	3.16	40.58	1.76	6.05	51.01	40,000

Percentage of self-inactivating (SIN)-Moloney murine leukemia virus (MLV), SIN-avian sarcoma-leukosis alpha-retrovirus (ASLV) and SIN-HIV integrations and random sequences targeting intergenic, transcription start sites (TSS)-proximal and intragenic regions, regions annotated as CpG islands, conserved non-coding (CNC) regions and transcription factor binding sites (TFBS). For all the comparison with random sites, we applied a two-sided Fisher’s exact test. * *p* < 0.01.

This analysis indicates that SIN-ASLV vector integrations have an almost random distribution in the human genome, with only a modest preference for genes and promoter regions compared to SIN-HIV and SIN-MLV vectors, suggesting entirely different modalities of target site selection.

We previously reported that SIN-MLV integrations are enriched around annotated CpG islands and conserved TFBSs and moderately enriched around mammalian, evolutionarily conserved non-coding sequences (CNCs) [14,25,26]. SIN-ASLV integrations were found associated with these genomic features at almost a random frequency, as observed for SIN-HIV integrations, and at a much lower frequency compared to SIN-MLV integrations (CpGs: 2.84% *vs.* 17.68%; TFBSs: 55.70% *vs.* 69.95%; CNCs: 5.49% *vs.* 8.42%, *p* < 0.01 in all cases) (Table 1), suggesting again that SIN-ASLV integrations have no obvious association with functional genomic elements.

We then looked at the tendency of the three types of retroviral vectors to target repetitive elements, by blasting both single- and multiple-match sequences to the UCSC RepeatMasker database and by annotating repetitive elements directly targeted by each integration site. Interestingly, only SIN-ASLV integrations were associated with repetitive elements with an almost random frequency (51% *vs.* 50%), while both SIN-MLV and SIN-HIV integrations were significantly under-represented in repetitive regions (37% and 45%, respectively, *p* < 0.01) (Table 2). By looking at the different classes of repetitive elements, we found that all three vectors have a slightly higher preference to integrate in short interspersed nuclear elements (SINEs) compared to random controls (17% to 20% *vs.* 15%), probably as a consequence of the fact that SINEs are often located in transcribed regions and contain PolII promoters [30,31]. On the contrary, integrations in long interspersed nuclear elements (LINEs), long terminal repeats (LTRs) and other repetitive elements were under-represented or close to random (Table 2). Finally, integration in satellite elements was observed at a random frequency only for SIN-ASLV vectors (0.43% *vs.* 0.35%), while both SIN-MLV and SIN-HIV integrations were significantly under-represented in these regions (0.01% and 0.05%, respectively, *p* < 0.01) (Table 2).

**Table 2:** Integrations targeting repetitive elements in the genome.

Vector	Repetitive elements (%)	LINEs (%)	SINEs (%)	Satellites (%)	LTRs (%)	Others (%)
SIN-ASLV (8,899)	51.29	19.50	19.77	0.43	6.26	5.35
SIN-MLV (13,606)	37.75	10.96	17.26	0.01	5.09	4.42
SIN-HIV (32,964)	45.78	19.45	16.86	0.05	4.08	5.35
Random (40,000)	50.96	21.27	14.69	0.35	9.57	5.10

Percentage of SIN-MLV, SIN-ASLV, SIN-HIV integrations and random sequences targeting repetitive elements and the percentage targeting each specific element: LINEs, short interspersed nuclear elements (SINEs), satellites, LTRs and all the other elements.

Overall, these data indicate a remarkably random pattern of integration for the ASLV-derived vector, which shows none of the characteristic preferences of gamma-retroviruses and lentiviruses for genes and genetic elements associated with gene function and regulation.

### 3.2. SIN-ASLV Integration Is Not Associated with Epigenetically-Defined Functional Genomic Regions

Many studies have reported a strong correlation between MLV and HIV integration sites and distinct epigenetic markers in different cell types (reviewed in [9]). In human CD34^+^ HSPCs, MLV integrations are strongly associated with histone modifications marking transcriptionally active PolII promoters and enhancers, while HIV integrations correlate with epigenetic markers of active PolII elongation within transcription units [14,32]. We therefore investigated the association of SIN-ASLV integrations with defined epigenetic markers of functional genomic elements. Taking advantage of publicly available ChIP-Seq data in the genome of human CD34^+^ HSPCs, we analyzed the association of SIN-MLV, SIN-ASLV and SIN-HIV integrations with specific histone modifications defining active or poised PolII promoters, enhancers, transcribed regions and heterochromatin (H3K4me1, H3K4me3, H3K36me3, H3K27me3 and H3K27ac).

**Figure 2:**
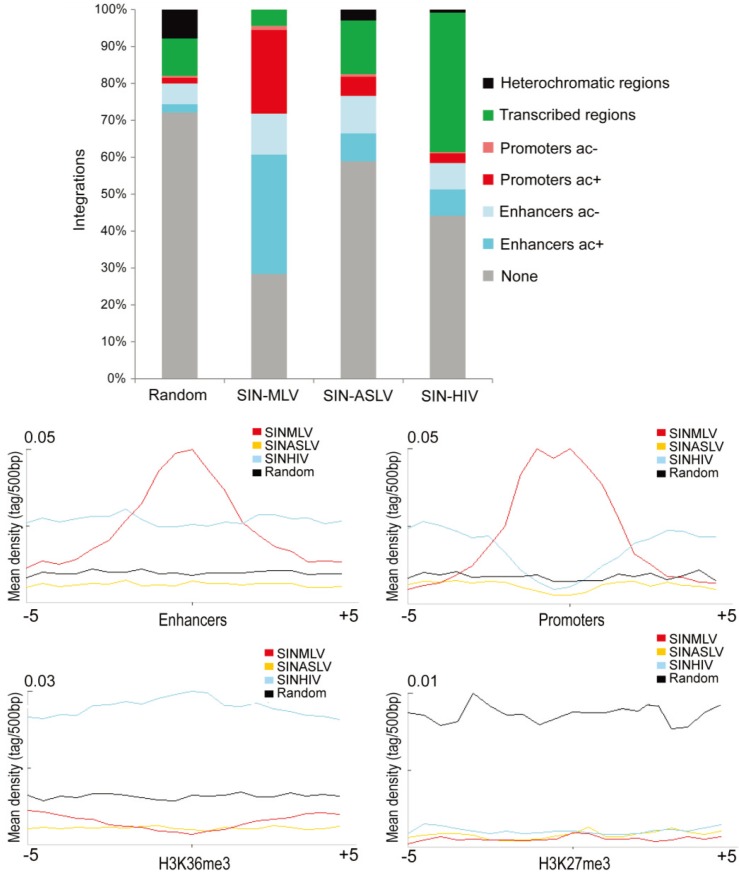
Association of vector integration sites with different epigenetically-defined chromatin states. (**a**) The percentage of integration sites associated with specific, epigenetically defined genomic regions for each vector type. Chromatin states are categorized on the basis of the combination of different epigenetic marks mapped by ChIP-seq in human HSPCs. Only integration sites that are unambiguously associated with one chromatin state were used for the analysis. (**b**) The mean densities of H3K4me1, H3K4me3, H3K36me3 and H3K27me3 ChIP-seq fragments in a 5-kb window around all SIN-MLV (red), SIN-ASLV (yellow) and SIN-HIV (light blue) integration sites and random sequences (black). ac: H3K27ac.

More than 60% and 70% of SIN-MLV and SIN-HIV integrations sites, respectively, were univocally associated with a defined chromatin state, compared to only 40% of the SIN-ASLV integration sites, a frequency very close to the 30% observed for random sequences. In particular, SIN-ASLV integrations were found around regulatory regions, *i.e.*, enhancer (H3K4me1^+^) and promoters (H3K4me3^+^), at a much lower frequency compared to SIN-MLV (10% *vs.* 37% in enhancers and 6% *vs.* 26% in promoters, respectively, *p* < 0.01), a tendency comparable to that observed for SIN-HIV (10% in enhancers and 4% in promoters) and slightly higher than that of the random sample (3% and 2%, respectively). Moreover, SIN-ASLV integrations were poorly associated with a marker of transcribed gene bodies (H3K36me3) compared to SIN-HIV (15% *vs.* 38%, *p* < 0.01). All three vectors were under-represented in heterochromatic regions marked by H3K27me3 compared to random sites, with the SIN-ASLV vector showing the highest association (Figure 2A). This analysis is in agreement with the associations observed at the level of DNA sequence and genomic annotations, and confirms the preference of SIN-MLV and SIN-HIV vectors for, respectively, regulatory sequences and transcribed regions and an almost random integration pattern for SIN-ASLV. The modest bias observed for SIN-ASLV integrations in DNase I hypersensitive regions compared to the random sample (Table S1) can be explained by a certain tendency to integrate in “open” chromatin regions, as observed for most retroviruses [14,16,33,34].

The differences between SIN-ASLV and the other two vectors in targeting defined chromatin regions are highlighted by plotting the average integration densities of each vector type around each histone modification. Indeed, we clearly observe a peak of SIN-MLV integration sites in a ±2.5-kb interval from epigenetically-defined enhancers and promoters, while the distribution of the SIN-ASLV and SIN-HIV integrations around these elements is similar to that observed for random sequences (Figure 2B). The quasi-random association of ASLV integrations in regulatory element predicts a much lower genotoxic risk compared to MLV-derived vectors, whose tendency to target active regulatory elements is at the basis of their propensity to cause insertional deregulation of gene expression [9]. Most (>70%) of the genes targeted by all three vectors in HSPCs are actively expressed (Figure S1), an expected finding, considering that retroviral target site selection is highly favored by an open chromatin state [14,16,33,34]. However, the SIN-ASLV vector targets the transcribed portion of active genes at a much lower frequency compared to HIV and is devoid of splicing signals, thus predicting a much lower risk to interfere with gene regulation at the post-transcriptional level [22,35,36].

### 3.3. SIN-ASLV Vector Integrations Are Not Clustered in the Human Genome

**Figure 3:**
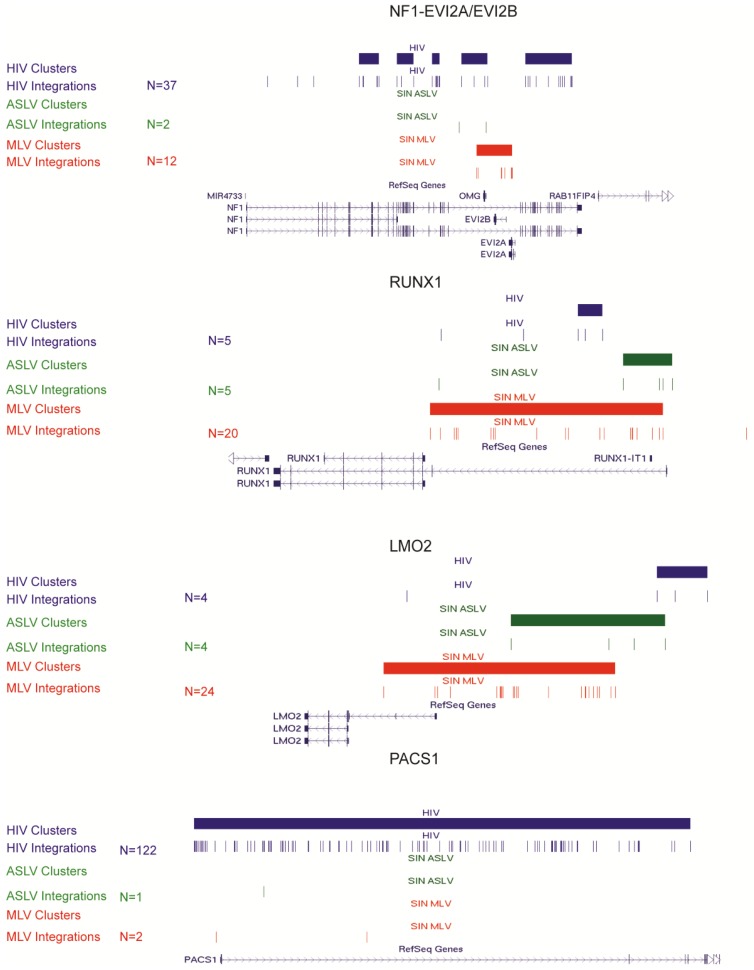
SIN-MLV, SIN-ASLV and SIN-HIV integration sites and clusters in CD34^+^ HSPC-specific loci. Distribution of SIN-MLV (red), SIN-ASLV (green) and SIN-HIV (blue) integration clusters (horizontal solid bars) and integrations (vertical marks) in the NF1-EVI2A/B, RUNX1, LMO2 and PACS1 loci, as displayed by the UCSC Genome Browser.

The integration profile of MLV- and HIV-derived vectors in the human genome is characterized by heavy clustering into integration hot spots, where MLV forms narrow clusters overlapping active regulatory elements and HIV larger clusters targeting a subset of transcribed genes, in both cases in a cell-specific fashion [14,15,16,24,37]. On the contrary, the SIN-ASLV vector showed no significant clustering when we applied a statistical definition of clusters adjusted to the numerosity of the sample [14], which for the SIN-ASLV dataset was three integrations in 53,920 bp. By this threshold, we were able to identify only 484 clusters, a significantly lower frequency compared to the 1,415 and 2,724 identified for the SIN-MLV and SIN-HIV vectors, respectively (*p* < 0.01). Only 21% of all SIN-ASLV integrations are clustered, compared to 56% and 51% of SIN-MLV and SIN-HIV integrations, respectively (*p* < 0.01) (Table 3). Moreover, SIN-ASLV clusters are mostly made of few (three or four) integrations with few clusters containing up to nine integrations, while SIN-MLV and SIN-HIV clusters contain up to 37 and 122 integrations, respectively. From these data, it appears that, contrary to other retroviral vectors, SIN-ASLV integrations do not form hot spots of integrations in the human genome. Interestingly, when we looked at integration clusters at single genomic loci, we observed that the frequency of SIN-ASLV integrations at the typical MLV or HIV hot spots is very low and comparable to the frequency observed for random sequences. Figure 3 shows a comparison of the integration pattern of the three vectors in the NF1-EVI2A/B, RUNX1, LMO2 and PACS1 loci, four known hot spots for MLV or HIV integration. The same scenario is true for the EVI1/MDS1 (MECOM) locus and all other MLV or HIV integration hot spots (data not shown). The low frequency of SIN-ASLV integration in proto-oncogenes responsible for the severe adverse events observed in clinical gene therapy trials, such as LMO2, provide a further indication of its lower genotoxic profile.

**Table 3:** Clusters of integration sites in the genome of human HSPCs.

	SIN-MLV (13,097)	SIN-ASLV (8250)	SIN-HIV (31,827)
Clusters	1415	484	2724
Integrations in clusters (%)	56	21	51
Average cluster dimension	5.1	3.6	5.9

The number of SIN-MLV, SIN-ASLV and SIN-HIV clusters of integrations, the percentage of integrations in clusters and the average cluster dimension, calculated based on random sequences distribution in the genome. The threshold for cluster definition was defined at a *p*-value of <0.01 by a statistical algorithm that adjusts for the numerosity of the sample [14].

Although the SIN-ASLV integration profile shows none of the features typical of MLV- and HIV-derived vectors, it is not completely random and shows a general preference for euchromatic regions. It is now know that both MLV and HIV pre-integration complexes (PICs) are targeted to chromatin by a tethering mechanism involving the interaction of the viral integrase with host cell factors: the LEDGF/p75 chromatin component interacts with the HIV integrase and directs its integration into transcribed gene bodies [38,39], while the MLV integrase appears to bind to bromodomain-containing BET proteins specifically associated with acetylated histones around TSSs and active regulatory elements [40,41,42]. Although it is likely that ASLV also may adopt a tethering mechanism to direct its integration in favorable genomic regions, the details are unknown. The integration preferences uncovered by our analysis predict an interaction with a broader range of host cell factors, which tether the PICs to open chromatin regions with unspecified or very subtle functional characteristics, thus leading to a more random profile characterized by the absence of hot spots.

## 4. Conclusions

Overcoming the genotoxic consequences of retroviral vector integration in the host cell genome is one of the major issues for the application of retroviral-based gene transfer in clinical trials. The strong preference to target TSSs, active regulatory elements or transcribed genes, together with the high frequency of clustering around hot spots, is a characteristics shared by all retroviral vectors currently used in clinical gene therapy. These characteristics are at the basis of the potential of retroviral insertion to deregulate gene expression at the transcriptional or post-transcriptional level, which has been observed to cause clonal expansion and contribute to neoplastic transformation in a number of cases (reviewed by [9]). Many efforts are being made to improve the safety of currently available retroviral vectors by removing the viral transcriptional control element and avoiding dominant, long-range acting enhancers in the transgene expression cassette. Retargeting vector integration has proven more difficult and was so far unsuccessful. No strategy is obviously perfect, and even a completely random integration machinery would not abolish the risk of inducing an insertional oncogenic mutation in the host cell genome.

Based on a genome-wide analysis of >8000 integration sites in human HSPCs, we show that a SIN-ASLV vector has a quasi-random integration pattern that privileges active chromatin regions, but is not associated with active regulatory elements, like MLV, or with transcribed genes, like HIV. More importantly, the SIN-ASLV vector showed no integration hot spots and no preferences for subsets of genes with a defined ontology or genes that were previously identified as being activated by retroviral insertion into tumors. Previous evaluations of ASLV-derived vectors in pre-clinical models proved its ability to sustain long-term transgene expression in murine and human hematopoietic progenitors and to correct the pathology in a mouse model of X-linked Chronic Granulomatous Disease (X-CGD), with no evidence of post-transcriptional interference [20,22]. Combined with the use of short-range or cell-specific transcriptional regulatory elements, an ASLV vector appears to offer a very safe profile and to be an ideal candidate for *ex vivo* gene therapy applications.

## Supplementary Files

Supplementary File 1 Supplementary Information (PDF, 60 KB)
