# Supplementary material for: Genome-Wide Analysis of Alpharetroviral Integration in Human Hematopoietic Stem/Progenitor Cells
Source: Genes (Basel). 2014 May 16;5(2):415–29. doi: 10.3390/genes5020415 (PMC4094940; doi:10.3390/genes5020415)
Supplement: Supplementary File 1 — Supplementary Information (PDF, 60 KB) [file genes-05-00415-s001.pdf]

Supplementary Information

Table S1. Integration distribution around DNase I hypersensitive sites in the genome of human HPSCs.

Vector	DNase I hypersensitive sites (%)
SIN-ASLV (8250)	18.26
SIN-MLV (13,097)	77.75
SIN-HIV (32,827)	12.21
Random (40,000)	9.00

Percentage of SIN-MLV, SIN-ASLV and SIN-HIV integrations and random sequences harboring a DNase I hypersensitive site in a ±1 kb interval.

Figure S1. Association of vector integration sites with CD34+ HSPCs gene expression.

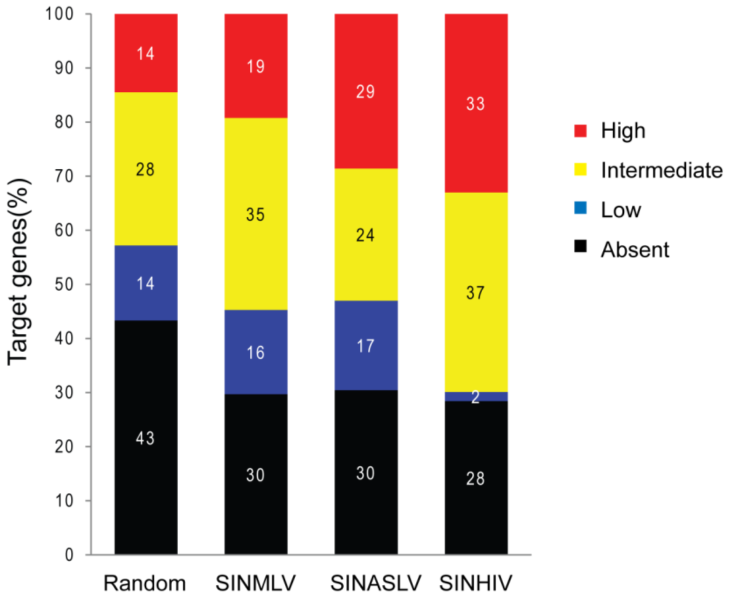

Histogram distribution of expression values from an Affymetrix microarray (HG-U133 + 2.0) analysis of RNA obtained from cytokine-stimulated $CD34^+$ cells. Expression levels were divided into four classes: absent, low (below the 25th percentile of the normalized distribution), intermediate (between the 25th and the 75th percentile) and high (above the 75th percentile). The percentage distribution of the expression values of genes targeted by SIN-MLV, SIN-ASLV and SIN-HIV integration sites and random sequences.

© 2014 by the authors; licensee MDPI, Basel, Switzerland. This article is an open access article distributed under the terms and conditions of the Creative Commons Attribution license (http://creativecommons.org/licenses/by/3.0/).
